# Cure of Encysted Dropsy by Exciting Irritation in the Sac

**Published:** 1846-03

**Authors:** 


ILLINOIS

MEDICAL & SURGICAL JOURNAL.

VOL. II. MARCH, 1846. NO. 12.

Cure of Encysted Dropsy, by exciting Irritation in the Sac.—Three cases of this disease, cured in the above mode, are related in the New York Journal of Medicine for November, 1845, by Edward H. Dixon, M. D., which, although wanting in details necessary to form a certain judgment of their character, are yet interesting at the present time, when the propriety of the extirpation of such tumors is exciting so much attention.

The first case was that of a woman 40 years of age, who had been for a length of time laboring under a dropsy and thrice tapped. After the third tapping, "an infusion of the sliced and dried fruit of the common persimmon" was thrown in with a 10 oz. syringe, with a catheter attached. The abdomen was handled, and the infusion then allowed to escape. Great prostration, followed by reaction, succeeded, and in the course of a few days "the lungs became hydropic, and frequently in one hour a large pocket handkerchief would be saturated to dripping with water coughed up from the lungs." By passing chloride of lime and sulphuric acid under the nose this was "driven to the skin," and a profuse sweat established, and during three regular metastases, the quantity of water was regularly diminished. The patient recovered. This very unusual case presents numerous points of interest, but from imperfect details and vague expressions used, it is doubtful whether the disease was one of ascites or an encysted dropsy, we infer the former.

The second case is one of encysted dropsy of the left ovary. Tapping having been performed, there was a discharge of a mellicerous substance, which continued. Solutions of sulph. Tinc., sulph. copper, oak bark, and of iodine, were thrown in successively, and had the effect of diminishing the discharge. The opening, however, still remains, although the tapping was performed ten years since, the patient enjoying good health.

The third case was also an encysted dropsy of large size, which was cured by a rupture of the sac from violence, the patient falling down stairs.

Another entirely similar to this last is reported by our friend Dr. James P. White, in a late No. of the Buffalo Medical Journal, the disease disappearing from the effects of a fall.

In addition to these, there are a large number of cases on record of encysted tumors of the abdomen containing serum, fatty or gelatinous matter, hair, &c., which have inflamed and suppurated either from the effect of violence, or without perceptible cause, and either been readily cured or left only a fistulous opening. It then becomes a question whether the injection of stimulating fluid and keeping open the puncture may not be introduced as a general practice. That this may be done with impunity in a certain number of cases, is shown not only by those above given, but by many other facts we might mention. Thus having occasion not long since to tap for ascites, we injected 25 grains of hyd. potass in solution, but no symptoms of inflammation resulted. But on the other hand we are not to forget that these encysted tumors, when large, often, if not most frequently, prove fatal from inflammation developed in and around them. Cases of this kind have come under our observation, in some of which there were several attacks relieved by anti-phlogistic treatment, before that which proved fatal. Nor is the plan of puncturing and injecting stimulating fluids new, but whether practiced through the abdominal walls, the vagina or rectum, they have in a majority of cases proved fatal.

We have not room for the introduction of cases, but refer any who may be desirous of investigating the subject to the work of Boivin & Duges on the Diseases of the Uterus and its appendages.

